# IL-1β mediated nanoscale surface clustering of integrin α5β1 regulates the adhesion of mesenchymal stem cells

**DOI:** 10.1038/s41598-021-86315-x

**Published:** 2021-03-25

**Authors:** Stephanie A. Maynard, Ekaterina Pchelintseva, Limor Zwi-Dantsis, Anika Nagelkerke, Sahana Gopal, Yuri E. Korchev, Andrew Shevchuk, Molly M. Stevens

**Affiliations:** 1 Department of Materials, Department of Bioengineering and Institute of Biomedical Engineering, Imperial College London, London, SW7 2AZ UK; 2 Department of Medicine, Imperial College London, London, W12 0NN UK; 3 Present Address: Department of Pharmaceutical Analysis, Groningen Research Institute of Pharmacy, University of Groningen, Groningen, The Netherlands

**Keywords:** Cell biology, Stem cells

## Abstract

Clinical use of human mesenchymal stem cells (hMSCs) is limited due to their rapid clearance, reducing their therapeutic efficacy. The inflammatory cytokine IL-1β activates hMSCs and is known to enhance their engraftment. Consequently, understanding the molecular mechanism of this inflammation-triggered adhesion is of great clinical interest to improving hMSC retention at sites of tissue damage. Integrins are cell–matrix adhesion receptors, and clustering of integrins at the nanoscale underlies cell adhesion. Here, we found that IL-1β enhances adhesion of hMSCs via increased focal adhesion contacts in an α5β1 integrin-specific manner. Further, through quantitative super-resolution imaging we elucidated that IL-1β specifically increases nanoscale integrin α5β1 availability and clustering at the plasma membrane, whilst conserving cluster area. Taken together, these results demonstrate that hMSC adhesion via IL-1β stimulation is partly regulated through integrin α5β1 spatial organization at the cell surface. These results provide new insight into integrin clustering in inflammation and provide a rational basis for design of therapies directed at improving hMSC engraftment.

## Introduction

Human mesenchymal stem cells (hMSCs) have been well described in their ability to modulate the immune response to repair tissues^1–3^, and thus show great therapeutic promise for several clinical situations. For example, they are widely researched for use in regenerative medicine involving repair or replacement of damaged tissues and as therapeutic biological vehicles to deliver gene therapies or drugs to many tissues in the body, whilst evading immune attack^4–7^. Despite in vitro evidence demonstrating their immunomodulatory activities, utilizing hMSCs clinically still remains challenging as they are often rapidly cleared, preventing them from carrying out their desired therapeutic effects^8,9^. Studies of the complex process of hMSC adhesion and migration to sites of inflammation are important for directing treatments towards damaged tissue and improving cell engraftment strategies. Specifically, as the location and density of individual molecules at the nanoscale dictates ligand binding and cell signaling, understanding the molecular processes underlying hMSC adhesion is of great clinical interest.

hMSCs are activated by pro-inflammatory cytokines, such as IL-1β, TNF-α and IFN-γ in a process termed ‘licensing’^10^. These cytokines are released from damaged tissue generating a concentration gradient, which directs the migration and adhesion of hMSCs to sites of injury^2,11^. IL-1β is a key inflammatory cytokine in the activation of hMSCs, expressed abundantly by many tissues of the body following injury, where it can induce its own expression in an autocrine manner leading to fast amplification of inflammation^12^. hMSCs exposed to IL-1β mount an anti-inflammatory response within 24–48 h^13–15^. Pre-treatment of hMSCs with IL-1β has been shown to enhance the efficacy of their engraftment in the treatment of ulcerative colitis^14,16^. Further, exposure of hMSCs to IL-1β has now been adapted for clinical use in the treatment of rheumatoid arthritis^13,17^, where preconditioning optimizes the cells for the microenvironment they will experience when introduced to the patient. It is suggested that IL-1β treatment contributes to the enhanced immunosuppressive abilities of hMSCs following adhesion at sites of injury, yet how it brings about such adhesion remains unknown.

Integrins are transmembrane receptors, that translate chemical and mechanical signals in a bidirectional manner across the plasma membrane to control cell–matrix adhesion and migration^18–21^. Clustering, or lateral association, of integrins at the plasma membrane forms a key component of integrin signaling and underpins migratory cell behavior due to their dynamic linkage to the cytoskeleton at focal adhesion (FA) complexes^22,23^. Ligand spacing modulates integrin activation, where 60–70 nm is the critical limit beyond which the cell does not recognize the integrins as clustered^24^. Studies have shown that cell motility can be regulated by varying integrin ligand spatial presentation on nano-patterned glass surfaces^25^. Changes in integrin clustering occurring at the nanoscale, can hence dictate cell behavior. Integrins have been implicated in the directed migration of hMSCs to sites of injury^11,26^. It was demonstrated that integrins can regulate both attachment and survival of hMSCs via adhesion to certain extracellular matrix (ECM) biomolecules^27,28^. One study established that ECM binding by integrin α5β1 directed migration of hMSCs in an α5β1-dependent manner^29^. The enhanced adhesion of hMSCs in inflammatory environments is suggested to occur via activation of surface integrins, specifically implicating α5β1 due to the fact it binds the ECM molecule fibronectin found in most tissues^2,30,31^. A recent study indicated that hydrogels with tethered ligands that bind specific integrins enhanced hMSC survival and engraftment by modulating their cytokine production and gene expression of factors associated with immunomodulation^32^. Further, it has been proposed that IL-1β can directly bind integrin α5β1, thus enhancing agonist IL-1β activity^33^. Whilst it is known that integrin α5β1 is involved in hMSC adhesion in many tissues, and activating hMSCs with IL-1β can enhance their engraftment, the underlying mechanism regulating this enhanced adhesion is not known. Integrin clustering is a general mechanism driving cell adhesion, consequently we hypothesized that the cell surface availability and molecular organization of integrin α5β1 at the membrane of hMSCs is likely regulated in an inflammatory environment and is hence crucial to their ability to migrate towards and bind the ECM at sites of tissue damage.

In this study, we set out to definitively assess a link between integrin α5β1 and hMSC adhesion, and to investigate whether IL-1β regulates the nanoscale organization of the receptor. Using the super-resolution microscopy technique direct stochastic optical reconstruction microscopy (dSTORM), we quantitatively analyzed the plasma membrane distribution of the integrin α5β1 receptor on hMSCs in the presence of IL-1β. Our results reveal that IL-1β increases hMSC adhesion through increased focal adhesion contacts, and specifically by inducing receptor availability and clustering of α5β1 at the nanoscale. This study not only offers new insights into the effects of inflammation on integrin α5β1 clustering, but also offers a basis for rational design of therapies directed toward improving hMSC engraftment.

## Results

To study the effect of IL-1β on hMSC adhesion and integrin α5β1 spatial distribution we utilized recombinant human IL-1β to stimulate the hMSCs. A concentration of 10 ng/mL was chosen to match that used in in vivo experiments that demonstrated enhanced hMSC transplantation in the treatment of ulcerative colitis^14^ and numerous other in vitro experiments in the literature e.g.^15,34,35^, thus enabling our results to be compared to effects of IL-1β in other studies.

### hMSCs retain their phenotype when activated by the inflammatory cytokine IL-1β

**Figure 1:**
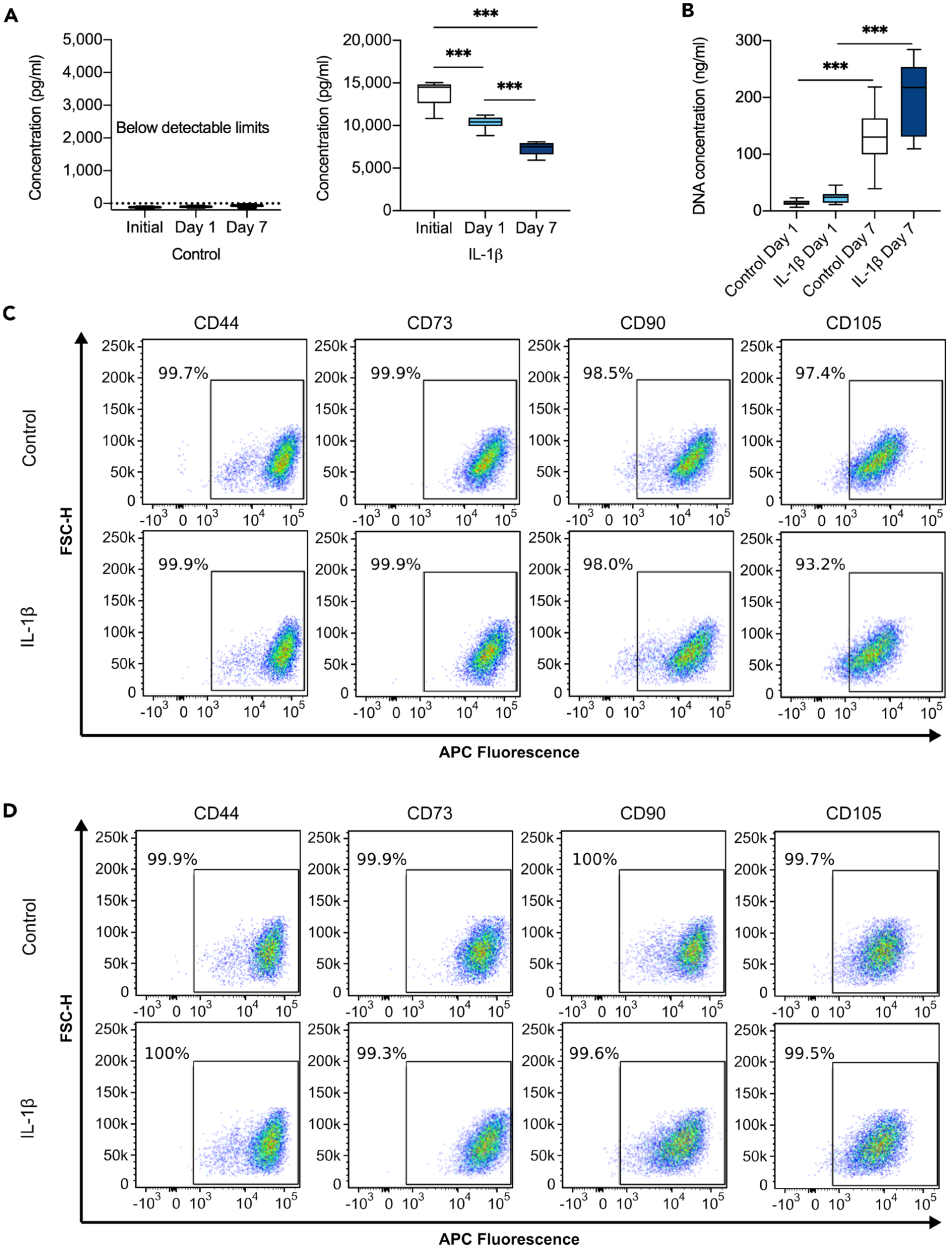
hMSCs retain their phenotype in the presence of IL-1β. **(A)** Quantification of the concentration of IL-1β in the cell medium upon initial addition of either control or IL-1β medium to cells and after 1 and 7 days in culture as measured by ELISA. N = 10 replicates for each condition. Parametric one-way ANOVA, Tukey multiple comparison test. **(B)** Quantification of DNA concentration of hMSCs after 1 and 7 days in culture. N = 27 replicates for each condition. Non-parametric Kruskal–Wallis test with Dunn’s multiple comparison test. ***p < 0.001. Box and whisker plots represent minimum to maximum. **(C,D)** Representative flow cytometry dot plots of forward scatter (FSC-H) versus APC fluorescence intensity for the cell surface markers CD44, CD73, CD90 and CD105 for control and IL-1β treated (10 ng/mL) hMSCs following **(C)** 1 day or (D) 7 days in culture. Gating strategy and analysis are in Supplementary Figure S1.

We first quantified and verified the presence of IL-1β in the cell culture media of IL-1β treated hMSCs using an ELISA, upon initial media addition and after 24 h (day 1) (Fig. 1A), the time point at which hMSCs are known to mount an anti-inflammatory response^13–15^. In control samples, no IL-1β was detected thus providing a faithful baseline control condition, for comparison to the IL-1β-induced inflammatory environment of the treated hMSCs. The concentration of IL-1β in the media of treated hMSCs was readily detected (Fig. 1A). A further time point after 7 days, with replenishment of control and IL-1β media at day 3, was also assessed and showed a considerable decrease in IL-1β in the cell culture media.

A prominent question is whether cultured hMSCs retain their phenotypic signature and thus their ability to carry out their functional roles in response to inflammation, or whether an inflammatory environment changes their phenotype. Characteristics of hMSCs include the ability to proliferate in culture and the presence of specific cell surface markers. Thus, the concentration of DNA was quantified after 1 day and 7 days in culture (Fig. 1B) as a measure of cell number. We found a substantial increase in the DNA concentration of hMSCs at day 7 compared to day 1, in both control and IL-1β conditions, demonstrating their proliferative function was not affected by IL-1β stimulation. To verify whether IL-1β stimulation affected their phenotypic signature, flow cytometry was carried out on the four characteristic hMSC surface markers; CD44, CD73, CD90 and CD105 (Fig. 1C,D). The gating strategy for flow cytometry is described in Supplementary Figure S1A, B. Almost 100% of the control and IL-1β treated hMSCs displayed each of the phenotypic markers, measured at day 1 (Fig. 1C and Suppl. Fig. S1C) and day 7 (Fig. 1D and Suppl. Fig. S1D) following stimulation, verifying they retain their phenotype following inflammatory stimulation with IL-1β, and for many days afterwards, at the least. Our data agree with a study that also confirmed inflammatory cytokines do not affect the phenotype of cultured hMSCs^36^.

### IL-1β enhances hMSC-substrate adhesion

**Figure 2:**
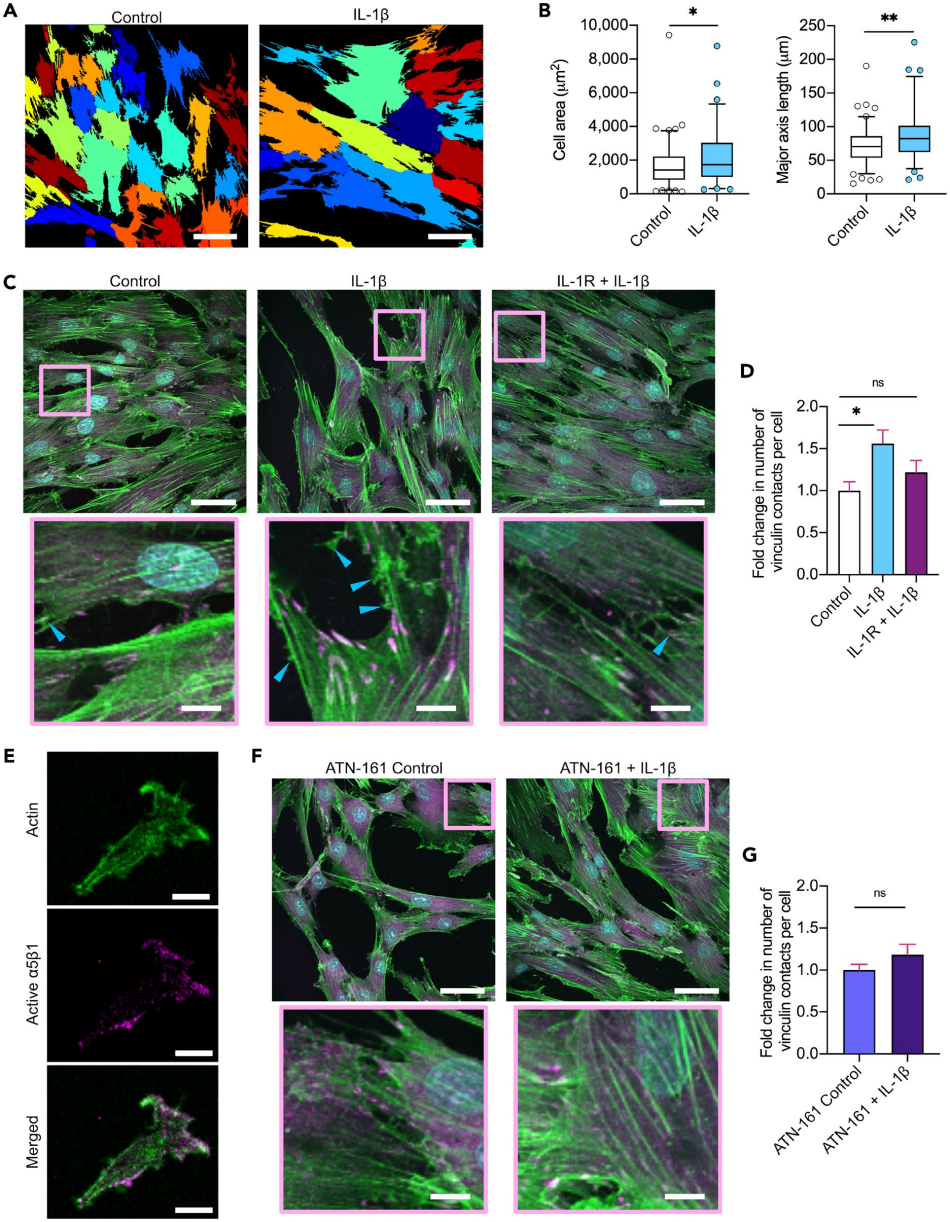
IL-1β increases hMSC adhesion via α5β1. **(A)** Representative cell area plots of control or IL-1β treated (10 ng/mL) hMSCs. Scale bar = 50 μm. **(B)** Quantification of hMSC area and length following IL-1β treatment. N = 73–101 (5 images per condition). Non-parametric unpaired two-tailed t-test, Mann–Whitney post hoc. **(C)** Representative confocal images of hMSCs in control media, treated with IL-1β, or IL-1β treatment after IL-1 receptor (IL-1R) blocking for 1 h, labeled for vinculin (magenta), actin (green), and nuclei (cyan). Areas highlighted with the magenta box are shown in the bottom row. Scale bar = 50 μm top row, 10 μm bottom row. Blue arrows indicate actin ruffles and filopodia. **(D)** Fold change in relative number of vinculin focal adhesions normalized to controls at day 1. N = 15–18 images total for each condition. Parametric one-way ANOVA, Tukey multiple comparison test. **(E)** Immunolabeling of actin (green) and active integrin α5β1 (magenta). Scale bar = 20 μm. **(F)** Representative confocal images of hMSCs treated with the α5β1 antagonist ATN-161 for 1 h followed by control media or IL-1β treatment, labeled for vinculin (magenta), actin (green), nuclei (cyan). Areas highlighted with the magenta box are shown in the bottom row. Scale bar = 50 μm top row, 10 μm bottom row. **(G)** Fold change in relative number of vinculin focal adhesions normalized to ATN-161 controls at day 1. N = 15 images total for each condition. Parametric unpaired two-tailed t-test. *p < 0.05, **p < 0.01, ns = not significant. Box and whisker plots represent 5th–95th percentile. Bar charts represent mean ± SEM.

Several studies have reported that IL-1β enhances engraftment of hMSCs^14,16,17^, although the mechanism is not yet clear. Adhesive area has been demonstrated to scale with adhesion strength of cells^37^, therefore we measured the area of hMSCs in control and IL-1β treated conditions (Fig. 2A,B). We show IL-1β causes an increase in cell spreading compared to controls (Fig. 2B). Further the major axis length of the cells was also increased when treated with IL-1β, suggesting the hMSCs also become more elongated upon such inflammatory stimulation.

FAs are micron scale complexes that form the main points of adhesion and traction between the cell and the underlying substrate, where it has been demonstrated that adhesion strength of cells to patterned protein exhibits an exponential increase with bound integrin numbers and vinculin recruitment^37^. Vinculin is a core FA protein crucially regulating integrin dynamics and clustering, and their link to the actin cytoskeleton, and is thus a marker of adhesion contacts^38–40^. To establish whether IL-1β increases hMSC presentation of adhesion contacts we analyzed the number of FAs per cell by quantifying vinculin immunolabeling following IL-1β treatment. Upon IL-1β stimulation, we saw an increase in the number of FAs per cell compared to controls (Fig. 2C,D). This number decreased when IL-1 receptors (IL-1R) were blocked by an antibody for 1 h, before addition of IL-1β. Additionally, a qualitative increase in actin membrane protrusions, stress fibers and ruffles, characteristic of elevated cell adhesion and migration, was visible in the presence of IL-1β (Fig. 2C). Combined, this data confirms that IL-1β stimulation enhances hMSC adhesion through increased contacts.

Integrins cluster at FAs, where they are activated leading to direct binding of the cell to the ECM. As integrin α5β1 has been linked to hMSC adhesion and migration, and IL-1β has been proposed to bind α5β1^33^, we carried out immunolabeling of integrin α5β1 utilizing a primary antibody (clone JBS5) that binds the agonist binding site which is only accessible when the receptor is in the active, extended conformation. This enabled visualization of the receptors actively involved in cell adhesion (Fig. 2E). As expected, active integrin α5β1 was found to be enriched at sites of tension of the actin cytoskeleton, confirming its role in cell adhesion.

Next we assessed if IL-1β could increase vinculin-positive cell adhesion contacts if α5β1 was blocked prior to inflammatory stimulation. Integrin α5β1 was blocked using the specific antagonist ATN-161 for 1 h before treating with control or IL-1β media, and vinculin immunolabeling was used to visualize the FAs. We found blocking α5β1 prior to IL-1β stimulation inhibited the increase in FA number when compared to respective controls (Fig. 2F,G). No difference was seen between controls and ATN-161 controls. Thus our results support the hypothesis that integrin α5β1 plays a role in IL-1β-induced hMSC adhesion, where downstream signaling between activated IL-1R and integrin α5β1 seems to play a role in the adhesion response.

The stiffness of the hMSCs was also assessed using scanning ion conductance microscopy (SICM) to measure cell resistance, however we found no difference in the stiffness of IL-1β treated hMSCs compared to control (Suppl. Fig. S2).

### IL-1β increases integrin α5β1 surface clustering

**Figure 3:**
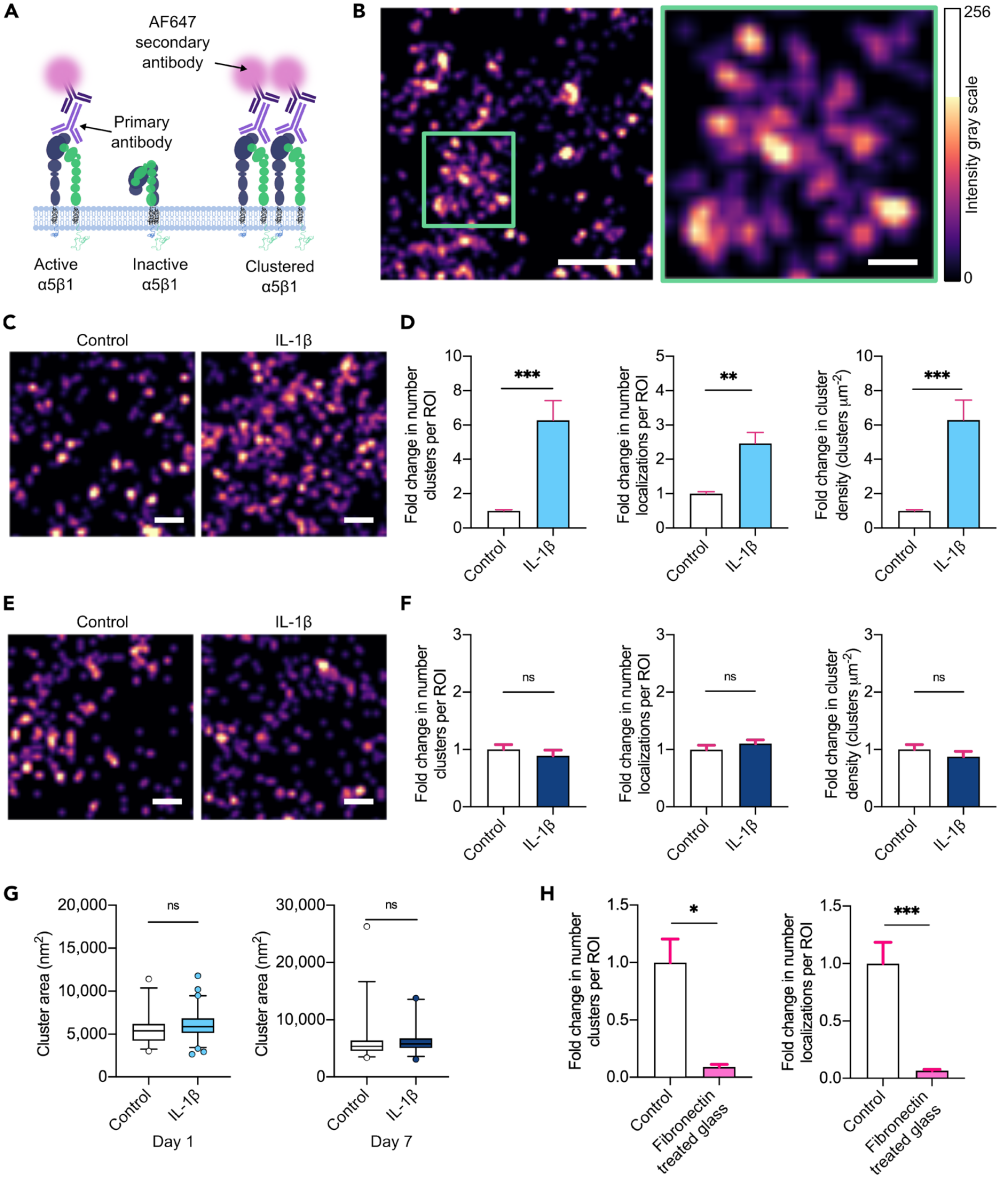
IL-1β induces α5β1 surface activation and clustering. **(A)** Schematic representing immunolabeling of active α5β1. **(B)** Representative dSTORM reconstructed image of integrin α5β1 clusters on the surface of an hMSC. Scale bar = 500 nm, zoom of green box = 100 nm. **(C)** Representative dSTORM reconstructed images of integrin α5β1 clusters on the surface of control and IL-1β treated (10 ng/mL) hMSCs following 1 day in culture. Scale bar = 200 nm. **(D)** Fold change in relative number of clusters, number of surface detections and cell surface cluster density, normalized to controls at day 1. **(E)** Representative dSTORM reconstructed images of integrin α5β1 clusters on the surface of control and IL-1β treated (10 ng/mL) hMSCs following 7 days in culture. Scale bar = 200 nm. **(F)** Fold change in relative number of clusters, number of surface detections and cell surface cluster density, normalized to controls at day 7. **(G)** Area of integrin α5β1 clusters at day 1 and 7. N = 3 independent experiments, 29–75 ROIs total for each condition. **(H)** Fold change in relative number of clusters and surface detections on hMSCs seeded on fibronectin-coated glass normalized to controls at day 1. N = 5–55 ROIs total. Non-parametric unpaired two-tailed t-test, Mann–Whitney post hoc. *p < 0.05, **p < 0.01, ***p < 0.001, ns = not significant. Bar charts represent mean ± SEM. Box and whisker plots represent 5th–95th percentile.

Given that blocking α5β1 inhibited IL-1β-increased hMSC adhesion, and clustering of integrins underpins cell adhesion at FAs, we wanted to test if the molecular organization of α5β1 receptors into clusters is implicated in IL-1β-induced hMSC adhesion. To investigate the nanoscale spatial organization of α5β1 we used dSTORM to quantitatively analyze the integrin receptors at the cell membrane. The capability of super-resolution imaging of exogenously labeled integrins has previously been demonstrated to quantitatively assess cluster sizes of ανβ3 in fibroblasts^41^. Quantification of integrin α5β1 was again carried out by utilizing the primary antibody (clone JBS5) that binds the active receptor^42–44^ (Fig. 3A). This enabled analysis of only the receptors that were made available for binding on the surface of the hMSC, and hence involved in cell adhesion. A secondary antibody conjugated with AlexaFluor647 was then added and dSTORM imaging performed to visualize the receptors. Reconstruction of the images by Gaussian fitting of individual fluorophore detections enabled visualization of the surface localizations (Fig. 3B). Density based spatial clustering of applications with noise (DBSCAN) based cluster analysis^45^ was used to identify the spatial membrane profile of α5β1. A high AlexaFluor647 photon intensity (Suppl. Fig. S3A) and a localization precision of 12.9 nm (Suppl. Fig. S3B) were measured, providing accurate nanoscale measurements.

Upon stimulation of hMSCs by IL-1β a visible increase in α5β1 receptor surface localizations could be seen at day 1 (Fig. 3C). The use of antibody binding in dSTORM cannot be used to interpret exact molecule numbers, due to steric hindrance of bound antibodies and the presence of multiple fluorophores on one secondary antibody. Therefore the fold changes in relative number of surface localizations, clusters and cluster density per membrane surface area were calculated by normalizing IL-1β results to controls. Our measurements reveal a 527% increase in mean number of α5β1 clusters, a 146% increase in the mean number of localizations and a 529% increase in the mean density of the α5β1 clusters within a given area of the plasma membrane following stimulation by IL-1β at day 1 (Fig. 3D). This means that IL-1β increases the number of receptors made available for binding at the cell surface, and moreover clusters these receptors, increasing the integrin adhesion sites. To determine if this increased membrane localization and clustering was sustained when IL-1β concentration decreased, we carried out dSTORM imaging and analysis at day 7. We found α5β1 surface localizations had returned to levels similar to control (Fig. 3E,F), which is consistent with the significant decrease in IL-1β present in the media by day 7, seen via ELISA (Fig. 1A). Our data therefore suggest that IL-1β stimulation of hMSCs leads to enhanced adhesion via increased nanoscale clustering of α5β1.

We further analyzed the cluster areas, and interestingly found a consistent cluster area for control and IL-1β stimulated cells at both time points, with a median cluster size of 5350–5850 nm^2^, corresponding to a cluster diameter of 82–85 nm (Fig. 3G). These values closely match the previously reported integrin cluster diameter of 80–120 nm on different stiffness substrates^41^. Thus our data imply cluster area may be tightly controlled, even in different biological environments.

To control for the activation of integrin α5β1, hMSCs were seeded on glass coverslips coated in fibronectin, and dSTORM was carried out. As the primary antibody to α5β1 binds the agonist site of the active receptor, prior hMSC binding to fibronectin precludes antibody binding^37^. Indeed, we found considerably decreased α5β1 localizations and clusters on the surface of hMSCs compared to controls, confirming diminished antibody labeling (Fig. 3H), validating the dSTORM quantification of active α5β1.

### IL-1β-increased α5β1 surface availability is due to activation of available integrins

**Figure 4:**
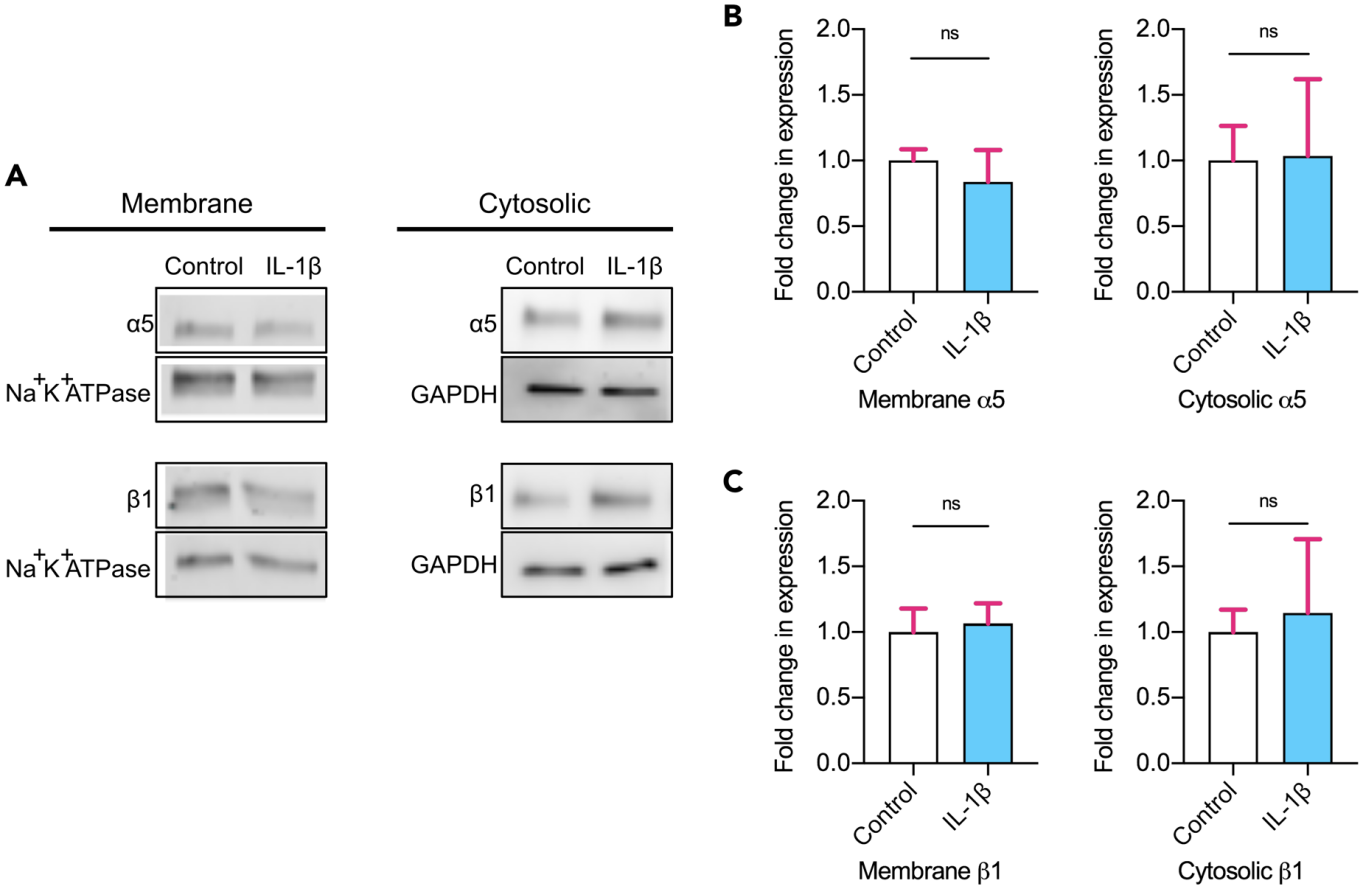
α5β1 total protein levels do not change. **(A)** Representative western blots of total protein membrane and cytosolic α5 and β1 integrin subunits in control and IL-1β treated hMSCs following 1 day in culture. Control and IL-1β samples were run on the same blot, with dividing lines delineating the samples from the housekeeping proteins used for normalization, probed on the same blot as the subunit protein of interest. **(B)** Fold change in α5 protein expression in the membrane and cytosol normalized to control. N = 6. **(C)** Fold change in β1 protein expression in the membrane and cytosol normalized to control. N = 6. Non-parametric unpaired two-tailed t-test, Mann–Whitney post hoc. ns = not significant. Bar charts represent mean ± SEM.

To verify whether the increase of α5β1 receptors at the membrane by IL-1β at day 1 occurred due to increasing the cellular protein expression of the receptor subunits or via activation of already available α5β1, total integrin protein expression levels were measured. We found no significant differences in the protein expression levels of the integrin subunits α5 or β1 at the membrane or in the cytosol (Fig. 4A–C and Suppl. Fig. S4). This led us to believe that total protein levels of α5β1 are unchanged in the presence of IL-1β, further confirming the fact that the primary antibody detects the active integrin conformation, and that the effect of IL-1β seen via dSTORM is due to increased activation of the available membrane integrins.

### IL-1β selectively induces integrin α5β1 clustering

**Figure 5:**
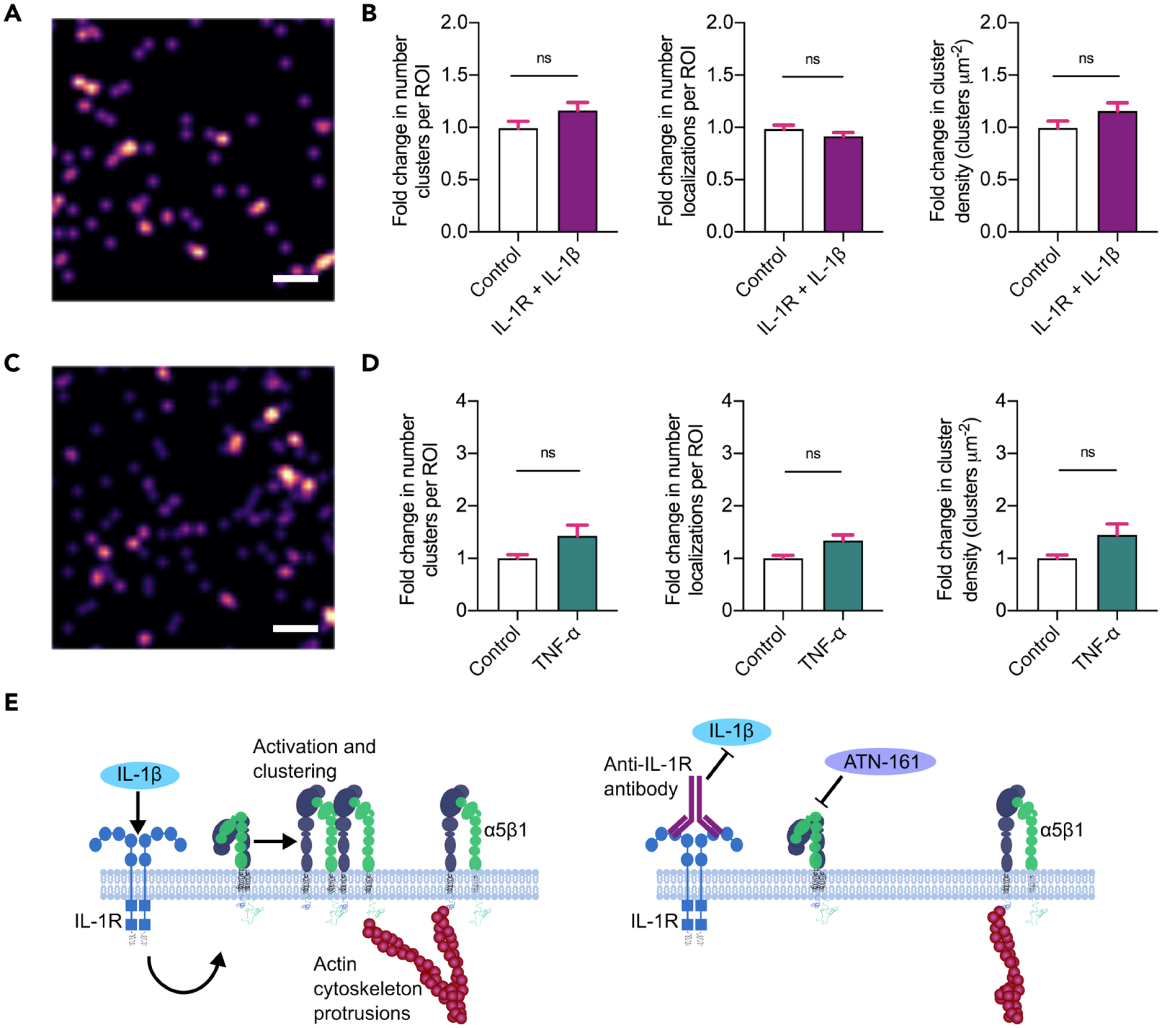
Blocking of IL-1R or TNF-α treatment prevents activation of surface α5β1 clustering. Representative dSTORM reconstructed images of integrin α5β1 clusters on the surface of hMSCs at day 1 following **(A)** IL-1β treatment (10 ng/mL) after IL-1 receptor (IL-1R) blocking for 1 h or **(C)** TNF-α treated (10 ng/mL). Scale bar = 200 nm. Fold change in relative number of clusters, number of surface detections and cell surface cluster density, normalized to respective controls at day 1 of **(B)** IL-1β treated (10 ng/mL) following IL-1R blocking for 1 h or **(D)** TNF-α treated (10 ng/mL) hMSCs. IL-1R + control data is plotted in Suppl. Fig. S4. N = 3 independent experiments, 50–100 ROIs total for each condition for IL-1R + IL-1β, 55–65 ROIs total for each condition for TNF-α. Non-parametric unpaired two-tailed t-test, Mann–Whitney post hoc. ns = not significant. Bar charts represent mean ± SEM. **(E)** Schematic of integrin α5β1 activation and clustering at the cell surface in the presence of IL-1β. This activation is prevented by prior antibody blocking of the IL-1R and does not occur in the presence of TNF-α.

To determine if the effect of IL-1β on increased surface α5β1 receptor numbers and clustering at day 1 was selective for the specific cytokine IL-1β, or merely due to the presence of an inflammatory environment, the IL-1R was blocked for 1 h prior to the addition of IL-1β and dSTORM was carried out as before. No visible differences in integrin labeling were seen (Fig. 5A). Quantitative analysis showed no change in α5β1 surface localizations or clustering when the IL-1R was blocked prior to IL-1β addition, compared to controls (Fig. 5B). Blocking of the IL-1R without the addition of IL-1β did not elicit any response compared to control (Suppl. Fig. S5). This illustrates that the effect of IL-1β on α5β1 clustering is specific, and occurs downstream of IL-1β binding to IL-1R. Additionally, this provides a further control validating the quantitative increase in active α5β1 in the presence of IL-1β as measured by dSTORM.

To further verify if the increase in α5β1 clustering was specific to IL-1β stimulation, the inflammatory cytokine TNF-α was added to the hMSCs at a concentration of 10 ng/mL, replicating the concentration of TNF-α used on hMSCs in other studies^46,47^, and dSTORM carried out at day 1 (Fig. 5C). The results showed no increase in α5β1 surface localizations or clusters compared to controls (Fig. 5D). Taken together, our results clearly demonstrate that the presence of IL-1β selectively increases α5β1 surface activation and clustering (Fig. 5E).

## Discussion

In this study, we hypothesized that integrin α5β1 nanoscale clustering, via IL-1β stimulation, transduces signaling that alters the adhesion of hMSCs. The hypothesis was based on studies that demonstrated IL-1β priming of hMSCs could enhance their engraftment in vivo, and that the general mechanism of integrin clustering underlies cell adhesion sites. We therefore wanted to investigate the inflammatory activation of hMSCs by IL-1β at the nanoscale, to gain insight into the biologically essential role of these cells in adhesion at sites of injury.

The assumption that enhanced engraftment of stimulated hMSCs is due to increased adhesion was supported by the increase in cell spreading and FA complexes as a response to IL-1β stimulation. Vinculin is a core structural protein of FAs and thus enabled us to identify adhesion contacts on the surface of the hMSCs. The observed increase in FA numbers could be inhibited by blocking the IL-1R or by blocking the α5β1 receptors prior to inflammatory stimulation. Our data therefore provided evidence of a role for α5β1 in the adhesion response elicited by IL-1β.

Through quantitative super-resolution imaging we then established IL-1β specifically augments integrin α5β1 receptor numbers available for binding at the plasma membrane and induces their clustering. Due to the recognized role of clustering of integrins in cell adhesion and migration^24,25,48^, the observed nanoscale changes of α5β1 clustering likely underpins the increased adhesion of hMSCs. One study reported direct binding of IL-1β to integrin α5β1^33^, however our data suggest activation of α5β1 is likely to occur downstream of IL-1β binding to IL-1R. Prior blocking of IL-1R prevented an increase in vinculin adhesion contacts and prevented the increase in α5β1 localizations and clustering at the membrane. If IL-1β directly bound α5β1 and induced its clustering we would expect to have seen the same increase in clustering with and without IL-1R blocking. Future studies into the intracellular molecular pathway by which IL-1β increases α5β1 clustering are thus merited. Candidates of the pathway may include ERK and IL-1R associated kinase (IRAK) which are activated downstream of IL-1R signaling and have been implicated in the formation of FA complexes^49,50^. IL-1β has previously been shown to induce phosphorylation and re-organization of talin^51^, which is a major regulator of integrin clustering, via activation of such kinases.

We also highlight that integrin cluster size is conserved between basal and inflammatory situations. One study showed a conserved cluster diameter of 80–120 nm for integrin ανβ3 on substrates of different stiffness^41^. Here we demonstrate that integrin α5β1 exhibits the same cluster diameter (80–90 nm) in both control and IL-1β stimulated environments. This is particularly remarkable, as it suggests that integrin cluster size is not only regulated in normal physiological conditions, but also in pathologically simulated situations, and has major implications for cluster size of other integrins in different pathophysiological environments, making these findings particularly significant.

Interrogating how hMSCs respond to inflammatory stimuli reveals parameters that regulate their immunomodulatory activities. Activation of hMSCs by inflammatory factors has been shown to enhance engraftment of the cells^14,17^, but it was not known how this occurred and hence how it could be exploited therapeutically. Here we definitively show that one of the key hMSC activating cytokines, IL-1β, specifically increases α5β1 surface clustering leading to increased integrin adhesions, whilst TNF-α, another hMSC licensing cytokine, had no effect on the nanoscale distribution of the receptor. Both IL-1β and TNF-α have been demonstrated to exert chemotactic activity on hMSCs^52^, and are both produced early following inflammation onset, however our results suggest these cytokines may provoke exclusive mechanisms by which this migratory behavior is induced.

Facilitating hMSC engraftment and homing by improving hMSC binding to sites of injury, achieved through a deeper understanding of binding cues should improve in vivo cell survival. Only a handful of tissue engineering studies have attempted to incorporate defined spatial organization of ligands in order to affect cell behavior. For example, one study demonstrated that clustered ligands on an elastin-like electrospun fabric enhanced integrin ανβ3-dependent clustering and focal adhesions as a function of ligand density^53^. A separate study found specific spacing of fibronectin binding sequences in nanofiber hydrogels induced an increase in integrin α5β1 expression in endothelial cells^54^. A recent study showed hydrogels designed to bind integrin α2β1 toward the treatment of bone defects increased hMSC osteoblastic differentiation^32^, suggesting regulation of tissue-specific binding can influence hMSC reparative functions. Therefore, insights from nanoscale biology involving receptor numbers and their spatial organization can inform the design of bio-instructive materials^55,56^. We speculate future studies employing strategic placement of ligands to cluster α5β1, could trigger specific downstream signaling leading to improved hMSC adhesion and migration, enhancing their clinical efficacy.

In summary, this study affords important observations for the critical role of α5β1 in hMSC adhesion and our data provides evidence that nanoscale spatial organization of integrin α5β1 on the surface of hMSCs is sensitive to the inflammatory microenvironment. Exploitation of the clustering of this receptor might represent a so far unexplored mechanism to modulate hMSC adhesion in vivo, to prevent rapid clearance of hMSCs, enabling them to carry out their desired therapeutic effect.

## Methods

### Cell culture

Primary human bone marrow-derived mesenchymal stem cells from three donors (hMSCs; Lonza) were cultured under standard cell culture conditions (37 °C, humidified atmosphere, 5% CO_2_) and used at passage 3–4 in all experiments. hMSCs were expanded in TheraPEAK chemically defined mesenchymal stem cell basal medium or mesenchymal stem cell basal medium (Lonza), supplemented with mesenchymal stem cell growth medium (Lonza) and 1% v/v Antibiotic/Antimycotic (A/A; Life Technologies). Cells were grown to 80–90% confluency in T175 cell culture flasks (Corning), trypsinized in 0.05% v/v Trypsin–EDTA (1X) (Life Technologies) and seeded at a concentration of 20,000 cells/cm^2^ in alpha minimal essential medium Glutamax^-1^ (αMEM; Life Technologies) supplemented with 10% v/v mesenchymal stem cell grade fetal bovine serum (MSC-FBS; Life Technologies) and 1% v/v A/A in glass bottom dishes for all experiments (24 well glass bottom SensoPlates (Greiner Bio-One) for flow cytometry, ELISA, DNA concentration and western blots, or 8 well glass bottom μ-slides (Ibidi GmbH) for dSTORM and confocal, or 35 mm high glass bottom μ-dishes (Ibidi GmbH) for SICM). The glass was not treated as the cell culture medium provides all ECM-derived adhesion molecules found in most tissues, thus providing an unbiased adhesion of the hMSCs to the glass enabling analysis of the effect of IL-1β on endogenous integrin α5β1. After 24 h fresh media with 10 ng/mL human recombinant IL-1β (R&D Systems) was added to the wells (or for controls simply replacing the media with fresh media without cytokines). Experiments were then carried out at day 1 after IL-1β addition, or day 7 with all media for control and IL-1β conditions replaced again at day 3.

For IL-1R blocking experiments, 24 h following cell seeding, human-reactive rabbit anti-IL-1 receptor polyclonal primary antibody (Abcam) was diluted 1:100 in fresh media and applied to the cells for 1 h at 37 °C before removing, washing once with fresh media and replacing with either fresh media without cytokines (IL-1R control) or fresh media with 10 ng/mL IL-1β (IL-1R + IL-1β). For integrin α5β1 blocking experiments, 24 h following cell seeding, fresh media containing 1 μg/μL ATN-161 (Sigma) was applied to the cells for 1 h at 37 °C before removing, washing once with fresh media and replacing with either fresh media without cytokines (ATN-161 control) or fresh media with 10 ng/mL IL-1β (ATN-161 + IL-1β). For TNF-α treated cells, 24 h following cell seeding, fresh media containing 10 ng/mL human recombinant TNF-α (R&D Systems) was applied to the cells (or for controls simply replacing the media with fresh media without cytokines).

For fibronectin coating, glass was treated with human fibronectin (Sigma; 1 mg/mL) diluted to 50 μg/mL in Dulbecco’s Phosphate Buffered Saline (DPBS; Life Technologies) overnight at 4 °C. Wells were washed three times in DPBS before cell seeding.

### Enzyme-linked immunosorbent assay (ELISA)

The media from the hMSCs was collected at initial addition, day 1 and day 7 time points and snap frozen immediately in liquid nitrogen. Quantikine ELISA kits (R&D Systems) were used to determine the concentration of IL-1β in the cell media as per manufacturer’s instructions. Briefly, media samples were diluted 1:75 in the given sample buffer and added to the plate along with a standard curve and incubated for 2 h. The plate was then washed three times in washing buffer, followed by incubation with horseradish peroxidase-conjugated antibody to IL-1β for 1 h at room temperature. This was followed by washing three times in washing buffer and addition of the substrate solution for 20 min at room temperature before addition of the stop reagent. The absorbance signal was read on a SpectraMax M5 plate reader at 540 nm. The results from the standard curve were used to interpolate the cytokine concentration of each sample.

### DNA concentration

Cells were collected from the wells at day 1, freeze-thawed (between − 80 °C and 37 °C) three times to lyse. For each sample, 50 μL of sample was added to 50 μL TE buffer (provided in kit) and 100 μL of 200 times diluted Quant-iT PicoGreen dsDNA marker in a 96-well plate (Corning). DNA standard was diluted to 250, 200, 150, 100, 25, 2.5, 1.25 and 0.25 ng/mL and added to the plate. The samples were incubated for 5 min at room temperature and the fluorescence read on a SpectraMax M5 plate reader at excitation 480 nm and emission 520 nm.

### Flow cytometry

Cells were trypsinized, filtered through a 40 μm mesh to remove cell aggregates and centrifuged at 300 × *g* for 5 min. The supernatant was removed and the pellet was stained with a Fixable Viability Dye eFluor450 (Thermo Fisher Scientific) for 30 min at 4 °C for dead cell exclusion. The cells were then washed with DPBS, centrifuged at 300 × *g* for 5 min and incubated with 0.1–10 μg/mL of APC-conjugated primary antibodies; CD44, CD73, CD90 and CD105 or APC mouse IgG1 κ isotype control (BioLegend) diluted in 3% w/v bovine serum albumin (BSA; Sigma) in DPBS on ice for 30 min. The cells were then washed again with DPBS, centrifuged at 300 × *g* for 5 min, fixed with 4% v/v paraformaldehyde (PFA; Electron Microscopy Sciences) in DPBS and analyzed by flow cytometry. All measurements were acquired on the LSRFortessa (BD Bioscience) and analyzed using Diva v.8 (https://www.bdbiosciences.com/en-us/instruments/research-instruments/research-software/flow-cytometry-acquisition/facsdiva-software ) and FlowJo v.9 (https://www.flowjo.com) softwares.

### Preparation of direct stochastic optical reconstruction microscopy (dSTORM) samples

hMSCs were fixed in 0.3% v/v glutaraldehyde (GA; Electron Microscopy Sciences) in cytoskeleton stabilization buffer (10 mM MES buffer pH 6.1, 150 mM NaCl, 5 mM EGTA, 5 mM Glucose, 5 mM MgCl_2 _)^57^, with 0.25% v/v Triton X-100 (Sigma) for 5 min, then fixed in 3% v/v GA in cytoskeleton stabilization buffer for 10 min. The cells were then treated with 0.1% w/v NaBH_4_ (aldehyde quenching to reduce background) in DPBS for 10 min, rinsed 1 × in DPBS followed by two more washes in DPBS for 10 min each. Cells were then blocked in 3% w/v BSA in DBPS for 2 h at room temperature, incubated with α5β1 primary antibody (mouse monoclonal, clone JBS5, 1:2000; Millipore) in 3% w/v BSA in DPBS for 1 h 30 min at room temperature, washed three times in DPBS, then incubated with AlexaFluor647 secondary antibody (goat anti-mouse IgG, 1:2000; Life Technologies) 1 h 30 min in 3% w/v BSA in DPBS. The cells were washed a further three times in DPBS followed by post fixation with 2% v/v PFA in DPBS for 10 min followed by a further three washes with DPBS.

### dSTORM image acquisition

The hMSCs were imaged in 25% v/v VectaShield (Vector Laboratories) in glycerol (Sigma)^58^. First a drop of the 25% v/v VectaShield in glycerol was added to the cells and a 7 mm diameter glass coverslip (VWR) placed on top to reduce oxygen exchange. The lid of the 8 well glass bottom μ-slide was also replaced to further reduce available oxygen. Imaging was carried out on a Zeiss Elyra PS.1 AxioObserver Z1 motorized inverted microscope with an electron multiplying charge-coupled device (EMCCD) camera (Andor iXon DU 897), an alpha Plan-Apochromat 100x/1.46 NA immersion oil DIC VIS Elyra objective and a 640 nm solid-state laser (150 mW). ZEN black image software v.2012 (https://www.zeiss.com/microscopy/us/products/microscope-software.html) was utilized to acquire the movies. Images were captured with EPI illumination, in ultra-high power mode (PALM_uHP), in 16-Bit depth with a pixel size of 100 nm and an image size of 24.8 μm × 24.8 μm. AlexaFluor647 was excited at 640 nm with an exposure time of 50 ms per frame at 80% laser power with an EMCCD gain of 10% and the fluorescence emission was acquired over 15,000 frames. As hMSCs are much bigger than the field of view with a 100 × objective, images were taken with as much of the cell in as possible.

### Cluster analysis

The single-molecule localization data were analyzed using the versatile, open source software ThunderSTORM v1.2 plug-in (https://zitmen.github.io/thunderstorm/)^59^ for FIJI v.2017 (https://imagej.net/Fiji). Camera parameters were input (pixel size 100 nm, photoelectrons per A/D count 8.6, base level 414, EM gain 10). Default fitting parameters were used (wavelet-based filter, local maximum detection of single molecules, and integrated two-dimensional Gaussian fitting). Multi-emitter fitting was selected for reconstruction to correct for over-blinking as each secondary antibody was conjugated with 5 fluorophores. Post-processing involved drift correction by cross-correlation, followed by filtering for an uncertainty ≤ 15 nm and frames merged (maximum 10) to remove localizations blinking continuously across several frames from the same molecule, thus avoiding over-counting. Images were reconstructed as 2D average shifted histograms with a bin size of 20 nm corresponding to a 5 × magnification. Cluster analysis was carried out using the Clus-DoC script (https://github.com/PRNicovich/ClusDoC)^45^ with MATLAB v.2017. The exported data from ThunderSTORM were uploaded into Clus-DoC and 5 regions of interest (ROI) of 4 μm × 4 μm were randomly selected for each image. This was done to ensure no background coverslip was analyzed, but only the flat surface of the hMSCs. DBSCAN was then used with a minimum number of neighbors (*MinPts*) of 3 for cluster propagation within a radius (epsilon) of 30 nm, with a cluster defined as having 10 localizations or more.

### Confocal imaging

Cells were fixed and quenched in exactly the same way as for dSTORM imaging described above. Following blocking in 3% w/v BSA in DBPS for 2 h at room temperature cells were incubated with vinculin (mouse monoclonal, clone hVIN-1, 1:400; Sigma) or α5β1 (mouse monoclonal, clone JBS5, 1:2000; Millipore) primary antibody in 3% w/v BSA in DPBS for 1 h 30 min at room temperature, washed three times in DPBS, then incubated with AlexaFluor488 secondary antibody (donkey anti-mouse IgG (H + L) for vinculin, or AlexaFluor647 secondary antibody (goat anti-mouse IgG , 1:500; Life Technologies) for α5β1, 1 h 30 min in 3% w/v BSA in PBS. Cells were washed three times in DPBS followed by incubation with AlexaFluor555-phalloidin (1:500; Life Technologies) and DAPI nuclear immunolabel (1:1000; Sigma) for 45 min at room temperature in 3% w/v BSA in DPBS. Cells were washed a further three times in DPBS, post-fixed in 2% v/v PFA in DPBS for 10 min followed by a further three washes with DPBS.

To image the samples, a drop of 100% VectaShield was added to the cells and a 7 mm diameter glass coverslip (VWR) placed on top. Imaging was carried out on a Leica SP5 MP/FLIM inverted confocal microscope, using a HCX alpha Plan-Apochromat 63x/1.4–0.6NA immersion oil objective, 405 nm laser diode, 488 nm and 514 nm argon lasers (30 mW). Leica LAS AF software v.2.7.3.9723 (https://leica-las-af-lite.software.informer.com/4.0/) was utilized to acquire the images. Images were captured via sequential scanning with a line average of 6 and a frame average of 3 at exactly the same intensity (405 nm = 25% laser power and 619 gain, 488 nm = 31% laser power and 666 gain, 543 nm = 30% laser power and 975 gain) for all images of all samples so comparison of intensities could be made. Images were captured at a size of 246.03 μm × 246.03 μm.

For cell area analysis actin and DAPI images (obtained along with vinculin immunolabeling) were loaded into CellProfiler v.4.07 (https://cellprofiler.org/)^60^. Using the DAPI to identify the nuclei ‘primary objects’, the actin was then used to identify the cell area ‘secondary objects’ with default settings (propagation, minimum cross-entropy thresholding).

For vinculin focal contact analysis composite images were produced in the image analysis software FIJI. Using the image analysis software ICY v.6.3 (http://icy.bioimageanalysis.org/)^61^ and using the wavelet ‘spot detector’ plugin v.1.8.0.0 (http://icy.bioimageanalysis.org/plugin/spot-detector/)^62^ at scale 2, 100%, with size filtering 5–3000, the vinculin FAs were detected as individual ROIs in each image. Using the ‘convert to ImageJ’ plugin in ICY the ROIs were saved for each image. Each image with its corresponding ROIs were opened in the software FIJI and the ‘Analyze > Measure’ function was utilized to measure the number of vinculin focal adhesions per image. The DAPI channel was utilized to count the number of cells per image so that the number of vinculin focal adhesions per cell could be calculated.

### Scanning ion conductance microscopy (SICM)

Cell media was exchanged for 37 °C Leibovitz’s L-15 CO_2_ independent media (L-15; Life Technologies). New borosilicate glass pipettes (O.D. 1 mm, I.D. 0.5; Intracell) were pulled using a P-2000 laser puller (Sutter Instruments, CA), producing a pipette with an inner tip diameter of 100 nm. The pipettes were filled with DPBS before use. Ion current measurements were carried out using Axopatch 200B amplifiers (Molecular Devices). A bias potential of 200 mV was used for imaging and traces were analyzed using a pClamp 10 (Molecular Devices). Stiffness maps were generated by simultaneously recording three topographical images at progressively increased set points using an approach speed of 30 nm ms^−1^ as previously reported^63^. Briefly at every image point, first the standard topographical measurement (Z height) was acquired at set point 0.3–0.4% of ion current drop compared to the reference (maximum) current, as previously described^64^. At this set point only minimal stress in the range of 0.1–10 Pa is exerted by the nanopipette on the cell membrane. The nanopipette was further lowered to two consecutive set points of 0.6% and 0.3% and corresponding heights stored as separate image points before the pipette was moved to the next position in the lateral plane. Eventually each image had recorded differential height maps effectively representing sample strain as the pipette deformed the sample at two higher, compressive stresses. Cell stiffness measurements were obtained for each height map on the basis of stress and strain using SICM Image Viewer software v.2 (Custom home built software, https://pubs.rsc.org/en/content/articlehtml/2018/nr/c8nr03870h), where stiffness values are given as fN/μm.

### Subcellular fractionation and western blotting

hMSCs were trypsinized and centrifuged at 500 × *g* for 5 min, washed with ice cold DPBS and centrifuged again at 500 × *g* for 3 min. Extraction of the cytoplasmic and membrane fractions was carried out using a subcellular fractionation kit (Thermo Fisher Scientific) according to manufacturer’s instructions. To carry out the western blots 75 μL of protein for each sample was mixed with 25 μL 4 × Laemmli sample buffer (BioRad) and 2.5 μL β-mercaptoethanol and heated at 95 °C for 5 min. Samples were added to Mini-Protean TGX pre-cast gels (BioRad) with Precision Plus Protein dual color standard. The gels were run at 0.03 A for 1 h 30 min on ice in Tris/Glycine SDS buffer (BioRad). The protein was transferred to a PVDF membrane (BioRad) at 100 V for 30 min on ice in Tris/Glycine buffer (BioRad). The membrane was blocked for 1 h at room temperature in 5% w/v BSA in TBS-T buffer (BioRad) with 0.1% v/v Tween-20 (Sigma). The membrane was incubated at 4 °C with primary antibodies; rabbit anti-α5 or rabbit anti-β1 (Cell Signalling Technologies) at 1:1000 in 5% w/v BSA in TBS-T buffer for membrane and cytoplasmic fractions. Membranes were washed three times 10 min in TBS-T followed by incubation for 1 h at room temperature with secondary antibody IRDye 800 goat anti-rabbit (Li-cor) at 1:10,000 in TBS-T for membrane fractions and anti-rabbit IgG HRP-linked (Cell Signalling Technologies) 1:2000 for cytoplasmic fractions. Membranes were washed a further three times before imaging membrane fractions on the Li-cor Odyssey imaging system and cytoplasmic fractions on the UVP BioSpectrum imaging system. The membranes were then probed for loading/housekeeping controls. Primary antibodies; mouse anti-Na^+^K^+^ATPase (Abcam, 1:500) for the membrane fraction and mouse-anti-GAPDH (Santa Cruz, 1:1000) for the cytoplasmic fraction were incubated in 5% w/v BSA in TBS-T overnight at 4 °C. Membranes were washed three times in TBS-T and incubated for 1 h at room temperature with secondary antibody IRDye 700 goat anti-mouse (Li-cor) at 1:10,000 in TBS-T for membrane fractions and anti-mouse IgG HRP-linked (Cell Signalling Technologies) at 1:2000 in TBS-T for cytoplasmic fractions. Membranes were washed a further three times in TBS-T before imaging membrane fractions on the Li-cor Odyssey imaging system and cytoplasmic fractions on the UVP BioSpectrum imaging system. Images of the membrane fractions were analyzed with the Li-cor Image Studio Lite v.5.2 (https://www.licor.com/bio/image-studio-lite/) and images of the cytosolic fractions were analyzed using FIJI “Analyze > Gels”.

### Quantification and statistical analysis

All graphing and statistical analyses were carried out using the software GraphPad Prism v8 (https://www.graphpad.com/scientific-software/prism/). Data were tested for normality of distribution using D’Agostino-Pearson and Kolmogorov–Smirnov tests. A parametric one-way ANOVA with Tukey multiple comparison test for significance was used to analyze ELISA and IL-1β-treated focal adhesion data. A non-parametric Kruskal–Wallis with Dunn’s multiple comparison test for significance was used to analyze DNA concentration. A parametric unpaired two-tailed t-test was used to analyze flow cytometry data and ATN-161 focal adhesion data. Non-parametric unpaired two-tailed t-test with Mann–Whitney post hoc was used for all other analysis. Data is represented as bar charts with mean ± standard error of the mean (SEM) for fold change data, or box and whisker plots for measured values. *p < 0.05, **p < 0.01, ***p < 0.001, ns = not significant.

## Supplementary Information

Supplementary Figures.

## Data Availability

Raw data is available on request from rdm-enquiries@imperial.ac.uk.
