# Supplementary material for: IL-1β mediated nanoscale surface clustering of integrin α5β1 regulates the adhesion of mesenchymal stem cells
Source: Sci Rep. 2021 Mar 25;11:6890. doi: 10.1038/s41598-021-86315-x (PMC7994456; doi:10.1038/s41598-021-86315-x)
Supplement: Supplementary file 1 — Supplementary Figures. [file 41598_2021_86315_MOESM1_ESM.docx]

**IL-1β mediated nanoscale surface clustering of integrin α5β1 regulates the adhesion of mesenchymal stem cells**

Stephanie A. Maynard^1^, Ekaterina Pchelintseva^1^, Limor Zwi-Dantsis^1^, Anika Nagelkerke^1^‡, Sahana Gopal^1,2^, Yuri E. Korchev^2^, Andrew Shevchuk^2^, Molly M. Stevens^1^*

^1^Department of Materials, Department of Bioengineering and Institute of Biomedical Engineering, Imperial College London, London, SW7 2AZ, UK

^2^Department of Medicine, Imperial College London, London, W12 0NN, UK

‡ Current address: Department of Pharmaceutical Analysis, Groningen Research Institute of Pharmacy, University of Groningen, The Netherlands

*Correspondence: m.stevens@imperial.ac.uk

Supplementary Information


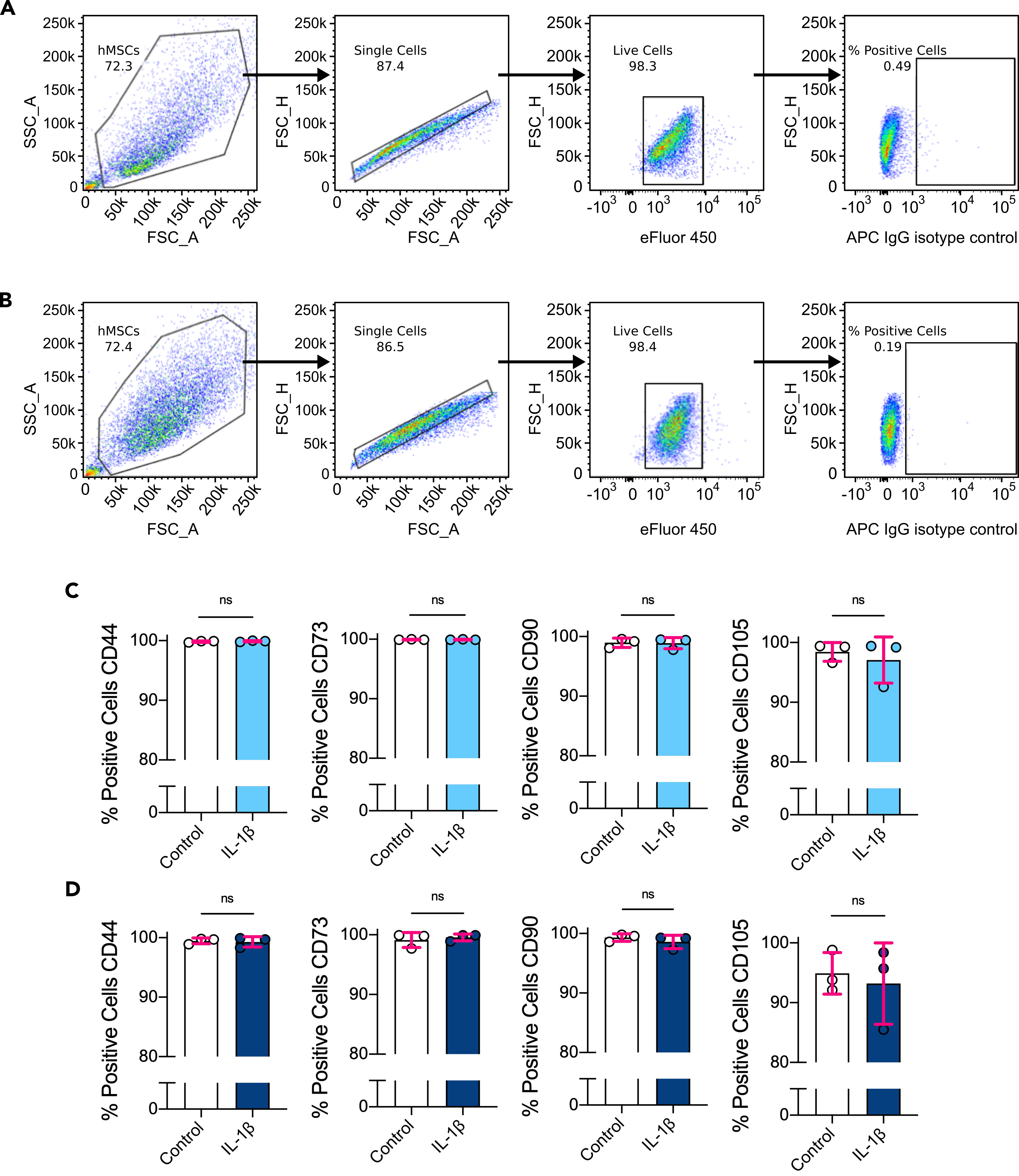


**Figure S1. Flow cytometry analysis of hMSCs treated with IL-1β.** Gating strategy for flow cytometry analysis following (A) 1 day or (B) 7 days in culture. The hMSC population was first gated (FSC_A vs. SSC_A) followed by gating for singlets (FSC_A vs. FSC_H). The single cells were further analyzed for their uptake of the live/dead eFluor 450 dye, taking only the live cells. APC mouse IgG isotype control was then used to set the gate for the APC-positive cells. Analysis of the percentage of cells positive for the cell surface markers CD44, CD73, CD90 and CD105, of control and IL-1β treated (10 ng/mL) hMSCs following (C) 1 day or (D) 7 days in culture. N = 3 independent experiments for all conditions. Parametric unpaired two-tailed t-test. ns = not significant. Bars represent mean ± SD.


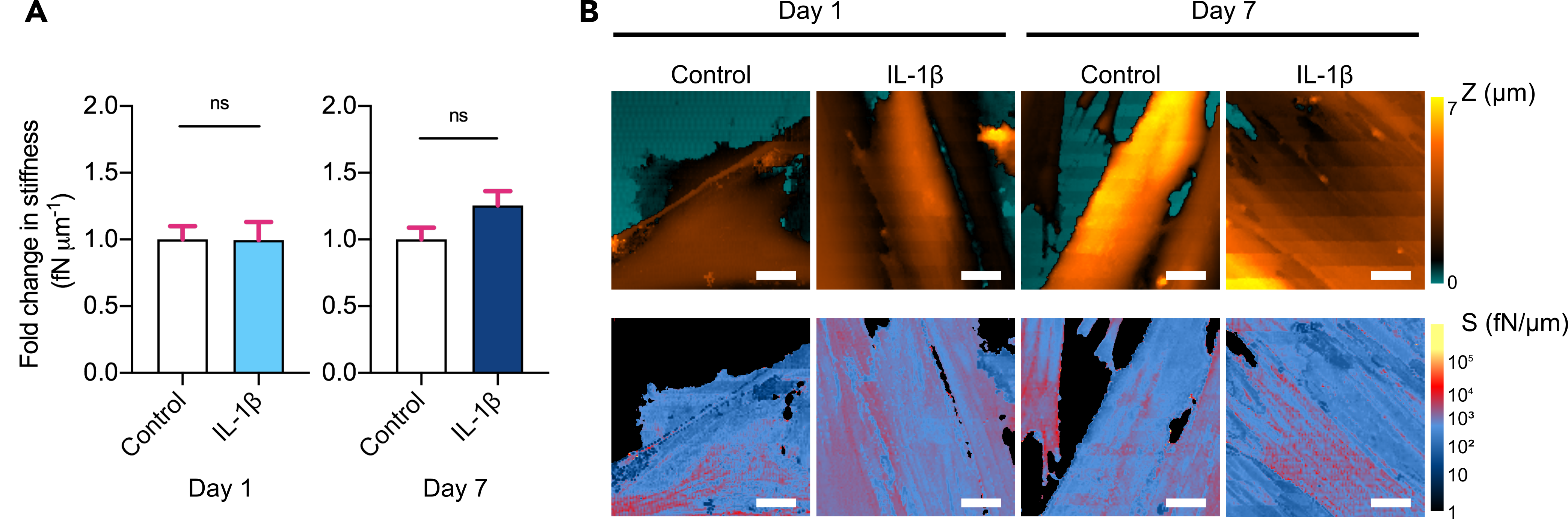


**Figure S2. Scanning ion conductance microscopy (SICM)** **stiffness analysis of hMSCs.** (A) Fold change in stiffness of IL-1β treated (10 ng/mL) hMSCs compared to controls following 1 day and 7 days in culture. N = 6, 17-22 ROIs total for each condition. Non-parametric unpaired two-tailed t-test, Mann-Whitney post hoc. ns = not significant. Bar charts represent mean ± SEM. (B) Representative height maps (top row) and stiffness maps (bottom row) of hMSCs. Scale bar = 10 μm.


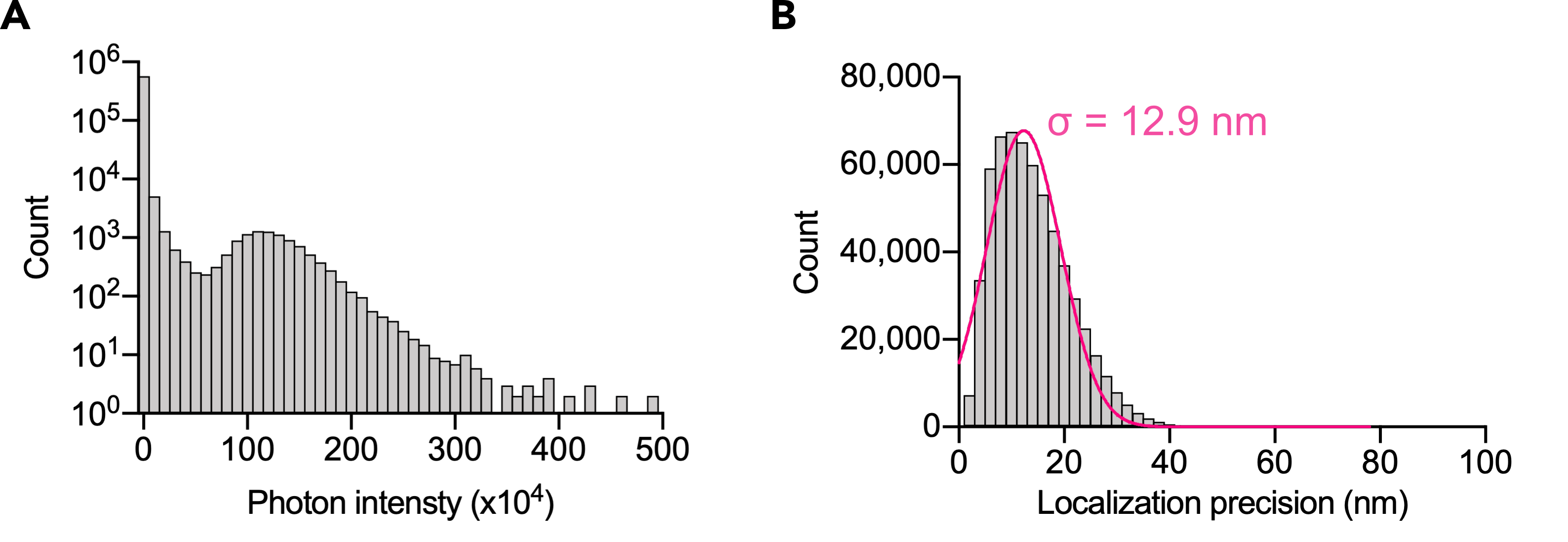


**Figure S3. dSTORM photon intensity and localization precision.** (A) Histogram of photon intensity of single AlexaFluor647 detections. (B) Localization precision (σ) of single AlexaFluor647 detections was measured, median = 12.9 nm.


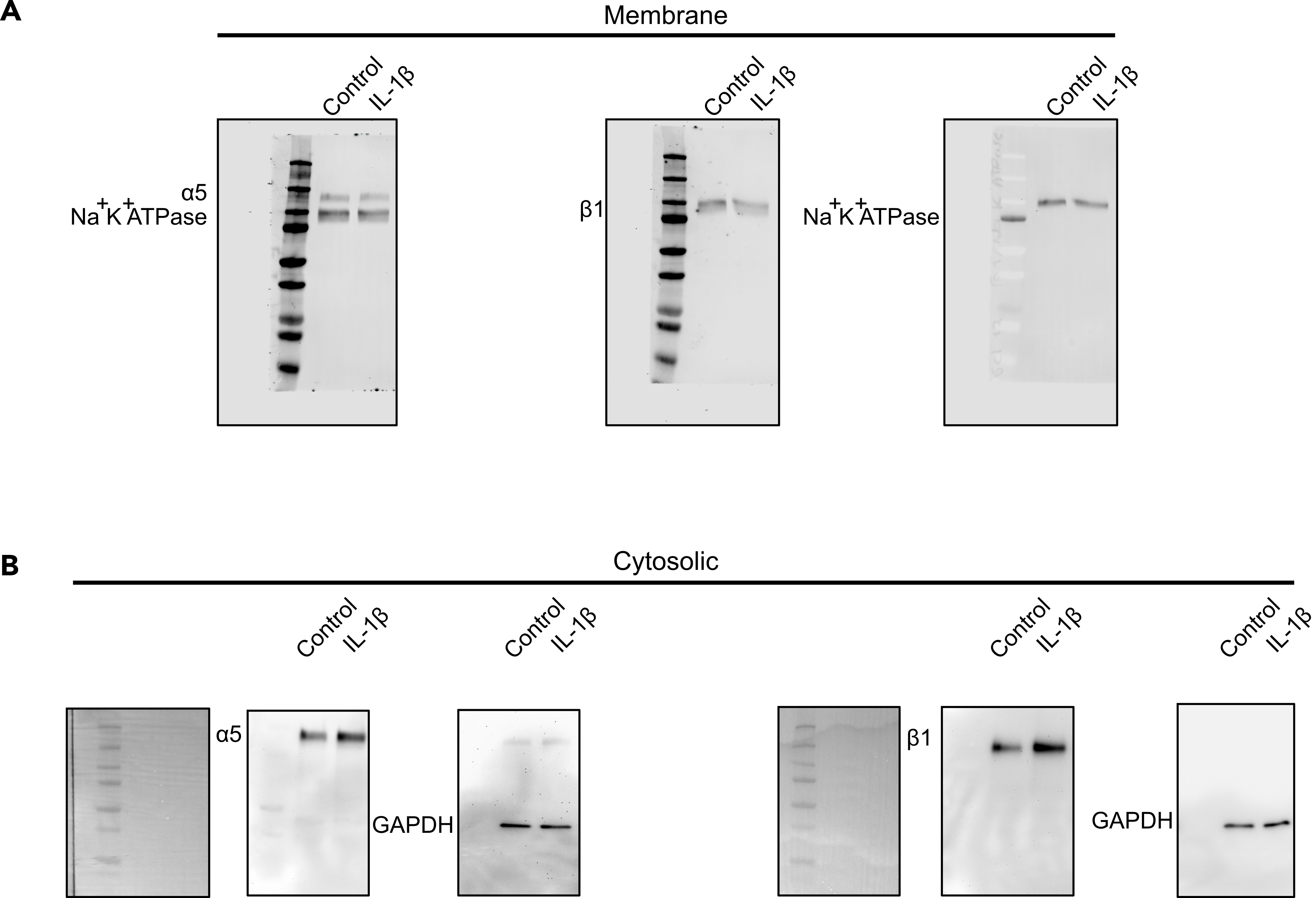


**Figure S4. Western blots of membrane and cytosolic α5 and β1 total protein levels.** Full length images of the representative western blots in figure 4. Membrane α5 and the membrane housekeeping gene Na^+^K^+^ATPase were imaged on the same blot. Membrane β1 was probed first, before Na^+^K^+^ATPase on the same blot. Cytosolic α5 and β1 were both probed first before the cytosolic housekeeping gene GAPDH on the same blot as the respective integrin subunits. The molecular weight ladder is not compatible with the HRP-linked secondaries utilized for cytosolic analysis so was imaged separately.


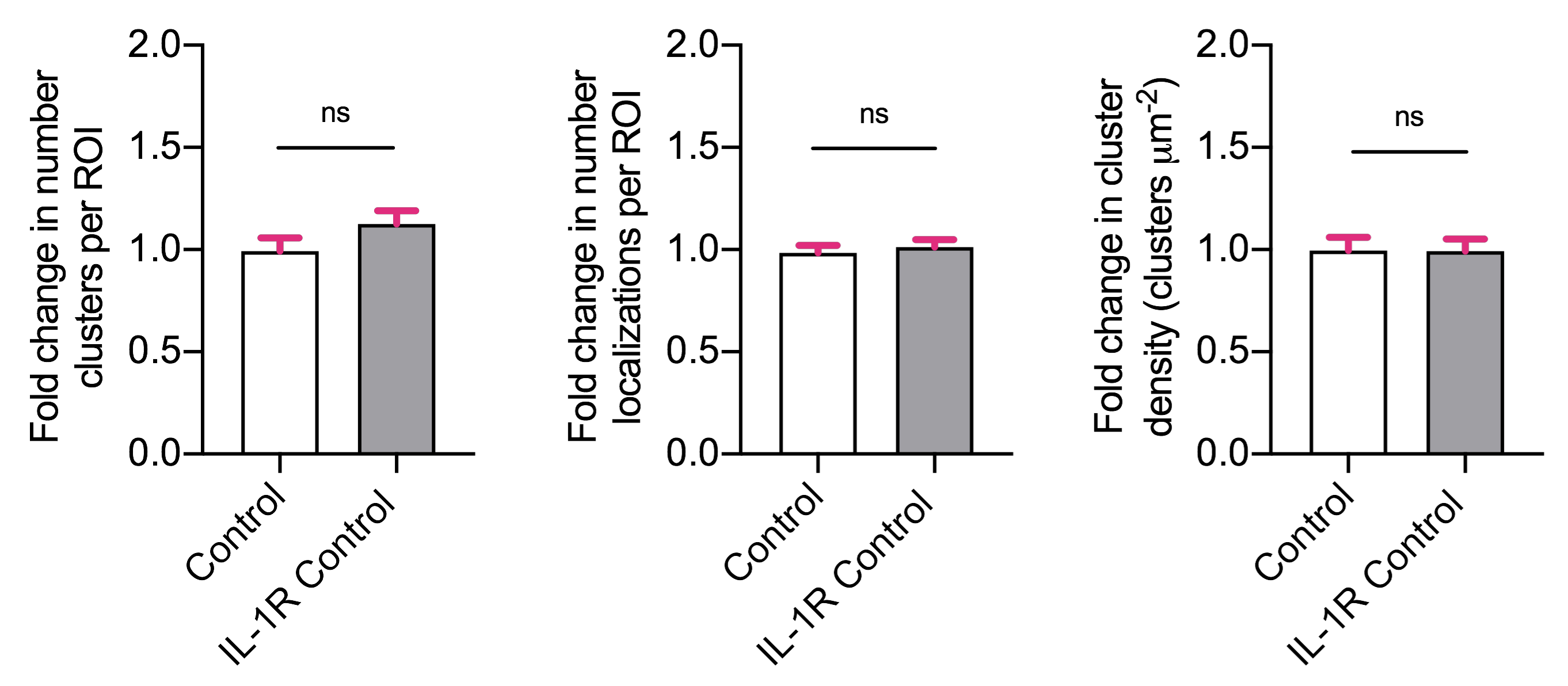


**Figure S5. Cluster analysis of IL-1R blocking controls.** Fold change in relative number of clusters, number of surface detections and density of clusters of integrin α5β1 on the surface of hMSCs following IL-1R blocking for 1 h and subsequent culture in control medium normalized to untreated controls at day 1. N = 3 independent experiments, 50-125 ROIs total for each condition. Non-parametric unpaired two-tailed t-test, Mann-Whitney post hoc. ns = not significant. Bar charts represent mean ± SEM.
